# How to Design Electronic Case Report Form (eCRF) Questions to Maximize Semantic Interoperability in Clinical Research

**DOI:** 10.2196/51598

**Published:** 2025-03-03

**Authors:** Eugenia Rinaldi, Caroline Stellmach, Sylvia Thun

**Affiliations:** 1 Core Unit Digital Medicine and Interoperability, Berlin Institute of Health, Charité - Universitätsmedizin Berlin, Luisenstr. 65, Berlin, 10117, Germany, 49 4930450543113

**Keywords:** case report form, CRF, interoperability, standard data model, data format, metadata, core data elements, data quality

## Abstract

Case report forms (CRFs) are the instruments used by research organizations worldwide to collect information about patients and study participants with the purpose of answering specific questions, assessing the efficacy and safety of medical products, and in general improving prevention and treatment in health care. To obtain significant research results out of the collected data, CRFs should be designed following the recommendations issued by regulatory authorities. However, we believe that semantic interoperability in CRFs has not yet been properly addressed. Within an international consortium comprising several COVID-19 cohorts, we scrutinized the questions included in the different CRFs with the purpose of establishing semantic interoperability across the different study data elements so that data could be merged and jointly analyzed. We realized that similar concepts were structured very differently across the different CRFs, making it hard to find and match the information. Based on the experience acquired, we developed 5 guiding principles on how to design CRFs to support semantic interoperability and increase data quality while also facilitating the sharing of data. Our aim in this viewpoint is to provide general suggestions that, in our opinion, should support researchers in designing CRFs. We conclude by urging authorities to establish an international coordination board for standards and interoperable clinical study data with competence in clinical data, interoperability standards, and data protection as part of a preparedness plan for future pandemics or other health threats.

## Introduction

Since the onset of the COVID-19 pandemic, we have witnessed the emergence of numerous studies worldwide aimed at deepening our understanding of SARS-CoV-2 infection and enhancing treatment strategies [1]. The urgency to produce meaningful research results in a relatively short timeframe has underscored the importance of efficiently merging data from diverse studies. However, differences in languages, formats, and terminologies often complicate the sharing and integration of data.

The European project ORCHESTRA [2], which sought to build a pan-European cohort of COVID-19 patients, confronted the significant challenge of harmonizing data from various cross-country studies [3]. Achieving interoperability among disparate datasets is critical not only for supporting ongoing research but also for bolstering preparedness against future global health crises. This aligns with the European Commission’s objectives under the European Health Data Space regulation [4], which emphasizes improving data accessibility and integration across member states.

Case report forms (CRFs) are essential tools employed by research organizations worldwide to gather detailed information from patients and study participants. These forms are designed to address specific research questions, evaluate the efficacy and safety of medical interventions, and ultimately advance prevention and treatment in health care.

A clinical study is designed to answer one or several research questions based on the analysis of data collected from patients that have been enrolled and are being observed or are partaking in specific interventions following the study protocol. CRFs, either in paper form or electronic format, are the instruments used to collect study data and form the basis of any subsequent statistical analysis. Electronic CRFs (eCRFs) are preferred over paper CRFs because data can automatically be stored in a digital format and immediately used, removing inaccuracies derived from the interpretation and transcription of handwriting. Additionally, if properly structured, the digital format intrinsically offers great potential for data objects to be more findable, accessible, interoperable, and reusable, in other words, more FAIR [5], than paper. The design of eCRFs is crucial for the outcome of a study [6]. Therefore, it should be optimized to enhance data quality and data interoperability.

Ideally, a standard operating procedure is established initially for designing eCRFs. Common recommendations for eCRF design include suggestions to reduce data entry errors and ambiguity in the interpretation of variables such as maximizing the use of coded questions and answer lists and minimizing the use of free text answers; using built-in consistency checks for admissible ranges and plausible date checks; facilitating data entry using branching logic strategies; specifying units of measurement (particularly for laboratory parameters, but also for vital signs, etc); adopting standard data formats; using (and reusing) published Common Data Elements, if available, and unambiguous temporal reference (eg, before or during infection).

Regulatory authorities and international expert organizations, such as the International Council for Harmonisation of Technical Requirements for Pharmaceuticals for Human Use and the Society for Clinical Data Management, have published detailed guidance on how to design CRFs, placing their focus on ensuring accuracy, utility, and poignancy of layout and content[7],[8] , [9]. However, the idea of designing CRF variables to facilitate standardization and reusability in the context of data interoperability has remained unaddressed by these expert institutions.

In the simplest setup, a study will be limited to 1 cohort, enrolling patients within one country, across several participating medical offices or hospitals. In more complex cases, patients are enrolled across several cohorts located in multiple countries. Regardless of the complexity of the study, interoperability and quality of data should always be considered high-priority objectives when designing the collection forms. By so doing, study results are more reliable, and collected data are ready for potential secondary use.

The COVID-19 pandemic, along with disease outbreaks caused by other viruses such as human monkeypox, Zika, and Ebola, has highlighted the need for international collaboration in terms of research. They have led to large-scale clinical studies conducted in the private sector and by public research consortia [10]. Multi-country and multi-cohort retrospective studies come with more challenges, as they generally need to combine data that were collected in different formats and, at times, different languages. This means that even when variables cover similar information, extensive transformation or translation activities are required before merging data can take place. This highlights the need for coherent study protocols across research groups and across countries, based on common formats and terminologies. Here is where data standardization and harmonization are the key to enabling quality data that can be merged easily without resource-heavy transformation activities, thus expediting analysis and gaining timely insights [11,12].

## Aim

Our aim in this viewpoint is to provide researchers with general suggestions in designing CRFs that, in our opinion, should support interoperability, reduce ambiguity, improve data quality, and facilitate data exchange across different systems.

## The ORCHESTRA Project

ORCHESTRA was funded by the European Commission during the COVID-19 pandemic and ran until the end of 2024. It aimed at creating a pan-European cohort of COVID-19 patients to study the disease, the efficacy of the treatment, and the long-term effects on general and fragile population as well as on health care workers. We were responsible for establishing interoperability within the European ORCHESTRA project. Partners in ORCHESTRA followed the goal to merge data from different clinical studies to generate new knowledge about multiple aspects of COVID-19 [3]. We examined over 3700 variables (comprised of questions and answers) with the objective of identifying similar information across studies that could be matched and analyzed jointly. This way, we compared the variety of approaches used across 7 different COVID-19 studies to investigate similar health care concepts. Within our project task, we associated international identifying codes from the most pertinent standard health terminologies to the questions and answers included in 7 clinical studies in ORCHESTRA. Ambiguous or complex wording found in the CRF variables needed to be evaluated as part of this mapping activity as well.

Based on our experience of being involved in large-scale national and international research projects, we believe that apart from ensuring accuracy and quality of collected data, CRF design should also maximize semantic interoperability. That way, time- and cost-efficient merging, analysis, and sharing of data can be facilitated. Our conviction is supported by the report published by the Joint Action Towards the European Health Data Space, which is a European initiative that developed principles for the secondary use of health data [13] and that places semantic interoperability as one of the operational objectives to achieve excellence of data quality.

To address the pressing need for streamlined data exchange and integration in clinical research, we have formulated 5 guiding principles that should be considered when designing CRFs. The principles address the need to harmonize data, unambiguously identify variables, associate clinical concepts with international identifiers, promote data quality, and enhance semantic coherence.

We believe that the application of the proposed principles would enhance semantic interoperability and support the exchange of information across different research groups.

## Five Guiding Principles to Enhance Interoperability of CRFs

Following our aim statement, we propose 5 guiding concepts aimed at increasing semantic interoperability of CRF data and quality of collected information.

### Harmonize Data

#### Concept 1: Creation or Reuse of Core Data Elements (CDEs)

Recurring information across clinical trials that are general in nature or specific to a specific disease should be identified and shared in a common format across the scientific community.

Data concerning demographics or clinical evaluation of patients, for example, is collected in many studies and should ideally be standardized to create a uniform format. Frequently collected disease progression and outcome information should also be identified and grouped into well-defined disease-specific core data elements (CDEs). CDEs can then be published for re-use by researchers worldwide.

Granted, the National Institutes of Health (NIH) have been focusing on developing CDEs for over 20 years. Yet, the adoption of CDEs is facing challenges. Reasons for low adoption by research projects, among others, are established data collection practices and ambiguous interpretations and implementations of health care concepts and CDEs.

The latter difficulty can, however be overcome by associating a common standard terminology to data elements to remove any ambiguity of meaning.

The increasing need to combine data in order to address global threats to life will eventually have to gain greater weight than maintaining localized, established practices.

National and international harmonization efforts such as the International Patient Summary (IPS) [14], the European Electronic Health Record Exchange Format [15], or the United States Core Data for Interoperability (USCDI) [16] should be considered to maximize reusability of data.

### Unambiguously Identify Variables

#### Concept 2: Use of Standards in CRF Metadata

Following the FAIR principles, metadata is of foremost importance for the quality re-use of information. Metadata should ideally include references to international terminology codes that unambiguously represent each concept. Whenever possible, all CRF variables should be handled as close-ended questions. In case of measurement or observations, Logical Observation Identifiers Names and Codes (LOINC) should be used. LOINC offers the possibility of defining data elements without ambiguity by specifying with one code all the details relative to the observation to be performed.

When no LOINC codes are available, other international terminologies should be considered, such as the National Cancer Institute’s Thesaurus for genomics data.

Qualitative answers should be restricted to defined (coded) value sets and identified with the appropriate terminology codes, such as those provided by Systematized Medical Nomenclature for Medicine–Clinical Terminology (SNOMED CT) [17]. The Anatomical Therapeutic Chemical Classification System can be used to describe drugs and chemicals, and the *International Classification of Diseases* to report diseases and disorders.

CRFs often include more complex questions that cover several informational components. That can include details about time, location, situation, etc. Hence, mapping several concepts to one semantic code can at times be difficult. This is a challenge that the postcoordination expression in SNOMED CT may help to solve in some contexts. However, integrating postcoordination into CRFs appears less feasible [18].

Terminology bindings proposed by national or international harmonization initiatives such as those mentioned in Concept 1 should be reflected in the metadata of the study data elements whenever possible. It should also be noted that the metadata could also include information on the format of the data. That can be achieved by mapping study elements to standards such as the Health Level Seven’s (HL7) Fast Healthcare Interoperability Resources (FHIR) standard or to the Observational Medical Outcomes Partnership Common Data Model.

The combined use of semantic and syntactic standards would further support interoperability.

### Associate Clinical Concepts With International Identifiers

#### Concept 3: One Concept at a Time (Unless It Is Part of a Questionnaire or Index)

The progressive adoption of FHIR by initiatives aiming to harmonize health data, such as IPS and USCDI, would suggest that information should follow the modular structure and be as precise as possible.

Hence, our second recommendation would be to only include one concept in a question. We have, for example, seen the following question in a COVID-19–related study CRF: asking enrolled patients whether they have had “Changes in or a loss of smell.” In this case, we propose splitting the question into 2 variables: “Changes in smell” and “Loss of smell.” With this approach, each variable could be represented by a specific semantic code. Additionally, splitting the question into 2 would also facilitate accurate analysis.

We acknowledge that this accuracy has to be balanced against the manageability of the length of a CRF. It should not lead to the creation of overly long CRFs, but rather to a focus on accuracy and key variables needed for analysis.

However, it is also important to note that at times some concepts which are included in questions could be removed without lowering accuracy of the question. For example, information like time points or target patients could be put as a header or as instructions within the CRF.

Of course, the ultimate decision on what constitutes the most relevant concepts to be included in the CRF questions always lies with the scientific group or the principal investigator or the sponsor.

### Promote Data Quality

#### Concept 4: Accurate Wording

Initiatives such as IPS and the USCDI provide guidance on the use of patient-related core data elements. However, often CRFs require the use of more study-specific variables. The wording of variables (questions and answers) should be carefully phrased to provide all and only the necessary information so that study nurses and respondents understand exactly what is asked. Precisely worded questions (and answer options) will increase the quality of data. This is to ensure that the data collected in response to these variables will be comparable and ready to be merged. In the case of laboratory examinations, the use of LOINC codes can be very useful because it automatically includes all the information necessary to remove ambiguity, that is, the methodology used, specimen type, or whether the expected result is qualitative or quantitative, unit information, etc.

If language translations of CRF variables are required, these should be meticulously performed. Additionally, a quality check on the wording and meaning of the translated variables is recommended and should be implemented, ideally by native speakers.

### Enhance Semantic Coherence

#### Concept 5: Answer Options Should All Be Semantically Coherent, and Units of Measures Clearly Stated and Identified With the Unified Code for Units of Measure Units

Another aspect of standardization relates to the answer options that are the second component of a CRF variable, after the question. It is important to maintain coherent semantic coding of answers as well. We recommend that, during the design of CRFs, semantic codes be used for mapping answers instead of assigning generic numeric identifiers that lack specificity. This would provide an unambiguous and reusable representation of the answer concept. Furthermore, coding would help the precision of the information and the quality of data by highlighting inconsistencies such as value set options not being semantically aligned.

For example, the question “What kind of swab test was performed?” should not include “throat” and “PCR” as answers in the same value set. In this case, the use of a terminology system like SNOMED CT clearly shows that the codes of the 2 concepts belong to different semantic categories (“body structure” and “procedure” respectively). Therefore, the question might be equivocal and could lead to unclear results.

In addition, in case of variables that describe quantitative (laboratory) measurements, such as “Dose of immunosuppressive medication taken per day” “Body weight” or “Glucose concentration measured” units of measure should be clearly stated and identified with Unified Code for Units of Measure codes [19,20].

## Summary and Considerations

Based on our experience in standardizing and harmonizing CRF variables from different protocols on COVID-19, we have presented five concepts aimed at improving CRF design and enhancing interoperability of clinical study data: (1) the creation of or (2) use of already existing standardized data objects can save time and help establish alignment and comparability with other research datasets, and (3) data quality can significantly be improved by paying attention to the fact that each CRF question and every answer option only contains 1 concept, (4) that variables are accurately phrased, and (5) that answer options are coherent, or in case of numerical results, that clearly defined units are included.

Probably, guidelines for reporting multiple concept codes in the metadata should be established, as this is a common occurrence in clinical research. In many cases, the possibility to code complex questions as coded questionnaires is very helpful. We believe it would be important to collaboratively address this difficulty of coding complex variables for the clinical study context.

The problem of not using interoperability standards is invisible to many researchers. That is because often, the advantages of such use become evident only when the need to merge data arises. Ideally, standards should be introduced already during the design phase of CRFs. Unfortunately, since the importance of data standards is still not adequately known, their implementation might be seen by some as a hassle that slows down or even limits the development of CRFs. In our opinion, a cultural change based on education and information in this field is needed.

It is necessary to abandon the idea of doing research in silos with data collected in incompatible formats by different research groups. Common data elements and their format should be identified, agreed on, and promoted by relevant national and international authorities. On the other hand, a scenario where clinicians are unwillingly responsible for standardization should be avoided, considering the already existing strain on their time and understandable gaps in expertise. New roles in health care are needed with expertise in digital medicine to enable interoperability of data and facilitate their integration within a wider eHealth ecosystem where data are being collected from different digital solutions and in a cross-country context.

The use of a common exchange format in combination with standard terminologies for study data elements in an eCRF would complete the interoperable data model of clinical research information. The innovative standard that is increasingly being adopted in the health care environment is HL7 FHIR. Thanks to FHIR’s innovative modular organization of information, a particularly efficient exchange of data is enabled. Its adoption in the clinical research environment is still low, but we expect this to change in the future. That is because the need to streamline activities and integrate information from electronic patient records or other medical devices into clinical research is progressively becoming more evident [21,22]. In fact, a dedicated HL7 working group is focusing on the design of FHIR resources to conduct clinical research more effectively [23].

In this context, as mentioned before, the European Commission has proposed a regulation for the European Health Data Space to support the interoperability of data in healthcare and in research. Forthcoming implementing acts will provide specifications for the exchange format of data to support cross-system and cross-border portability. In the United States, the Office of the National Coordinator for Health Information Technology promotes the USCDI [16], a set of health data elements divided into thematic classes to support information exchange. In general, collaborations to improve interoperability are fortunately increasing, including, for example, the Observational Health Data Sciences and Informatics (OHDSI) and the NIH concerning the maternal health data [24] or the employment of the Observational Medical Outcomes Partnership Common Data Model by the NIH research program “All of Us” [25]. Very important is also the OHDSI and European Medicines Agency collaboration in the project DARWIN [26], and the HL7 Vulcan project bringing together OHDSI and HL7 FHIR [27].

In conclusion, the COVID-19 pandemic has revealed to the broad community how important it is to quickly analyze large amounts of data, develop vaccines, and assess their safety and efficacy. We therefore need to facilitate the exchange of information in the context of global health challenges (including cancer, infectious disease, and rare diseases) and implement standardization of clinical study data collection. Additionally, the establishment of an international coordination board for standards and interoperable clinical study data with competence in clinical data, interoperability standards, and data protection should be part of a preparedness plan to face future pandemics and other health threats. This proposed coordination board, in coordination with ongoing international initiatives, could be instituted at the regional level and associated with large funding bodies and policy makers (ie, European Commission within the European Union and NIH in the United States) or at the pan-regional level, for example, as part of the World Health Organization.

## Abbreviations

CDE: core data element
CRF: case report form
eCRF: electronic case report form
FHIR: Fast Healthcare Interoperability Resources
HL7: Health Level Seven
IPS: International Patient Summary
NIH: National Institutes of Health
OHDSI: Observational Health Data Sciences and Informatics
SNOMED CT: Systematized Medical Nomenclature for Medicine–Clinical Terminology
USCDI: United States Core Data for Interoperability
